# Comorbidities of Patients With Idiopathic Pulmonary Fibrosis in Four Latin American Countries. Are There Differences by Country and Altitude?

**DOI:** 10.3389/fmed.2021.679487

**Published:** 2021-06-17

**Authors:** Mauricio Gonzalez-Garcia, Emily Rincon-Alvarez, Maria Laura Alberti, Mauricio Duran, Fabian Caro, Maria del Carmen Venero, Yuri Edison Liberato, Ivette Buendia-Roldan

**Affiliations:** ^1^Fundación Neumológica Colombiana, Bogotá, Colombia; ^2^Hospital María Ferrer, Buenos Aires, Argentina; ^3^Hospital Nacional Arzobispo Loayza, Lima, Peru; ^4^Hospital Belén de Trujillo, Trujillo, Peru; ^5^Instituto Nacional de Enfermedades Respiratorias Ismael Cosío Villegas, Ciudad de México, Mexico

**Keywords:** idiopathic pulmonary fibrosis, comorbidities, Latin America, altitude, pulmonary hypertension

## Abstract

**Background:** Comorbidities in idiopathic pulmonary fibrosis (IPF) affect quality of life, symptoms, disease progression and survival. It is unknown what are the comorbidities in patients with IPF in Latin America (LA) and if there are differences between countries. Our objective was to compare IPF comorbidities in four countries and analyze possible differences by altitude.

**Methods:** Patients with IPF according 2012 ATS/ERS/JRS/ALAT guidelines, from two cities with an altitude of ≥2,250 m: Mexico City (Mexico) and Bogotá (Colombia) and from three at sea level: Buenos Aires (Argentina) and Lima and Trujillo (Peru). Comorbidities and pulmonary function tests were taken from clinical records. Possible pulmonary hypertension (PH) was defined by findings in the transthoracic echocardiogram of systolic pulmonary arterial pressure (sPAP) >36 mmHg or indirect signs of PH in the absence of other causes of PH. Emphysema as the concomitant finding of IPF criteria on chest tomography plus emphysema in the upper lobes. ANOVA or Kruskal Wallis and χ^2^-tests were used for comparison.

**Results:** Two hundred and seventy-six patients were included, 50 from Argentina, 86 from Colombia, 91 from Mexico and 49 from Peru. There prevalence of PH was higher in Colombia and Mexico (*p* < 0.001), systemic arterial hypertension in Argentina (*p* < 0.015), gastro-esophageal reflux and dyslipidemia in Colombia and Argentina (*p* < 0.001) and diabetes mellitus in Mexico (*p* < 0.007). Other comorbidities were obesity (28.4%), coronary artery disease (15.2%) and emphysema (14.9%), with no differences between countries. There was more PH in the altitude cities than those at sea level (51.7 vs. 15.3%, *p* < 0.001). In patients from Bogotá and Mexico City, arterial oxygen pressure, saturation (*p* < 0.001) and carbon monoxide diffusing capacity (*p* = 0.004) were significantly lower than in cities at sea level.

**Conclusions:** In this study with a significant number of patients, we were able to describe and compare the comorbidities of IPF in four LA countries, which contributes to the epidemiological data of this disease in the region. The main results were the differences in comorbidities between the countries and more PH in the subjects residing in the cities of higher altitude, a finding that should be validated in future studies.

## Introduction

Idiopathic pulmonary fibrosis (IPF) is a specific form of chronic fibrosante interstitial disease, progressive, unknown-cause, which occurs mainly in older adults and is limited to the lung (1). Among idiopathic interstitial pneumonias is the most common, with an incidence of 3–9 cases per 100,000 and a prevalence of 18–20 cases per 100,000 (2–4). The natural history of IPF is of progressive decline in lung function, with an average survival of 3–5 years from diagnosis (2, 3, 5, 6).

Respiratory and non-respiratory comorbidities have been identified in IFP, some also associated with aging, being the most common are sleep apnea, pulmonary hypertension (PH) and gastroesophageal reflux (GER). These comorbidities affect patients' quality of life, can increase symptoms, contribute to disease progression and increase mortality (7, 8).

In Latin America there are no studies on the comorbidities associated with IPF. Taking into account the differences between the countries in terms of the prevalence of risk factors and comorbidities in the general population, we consider that there could be differences in the comorbidities of IPF. Additionally, in cities located at high altitude such as Mexico City (2,240 m) and Bogotá (2,640 m), due to the decrease in barometric pressure (PB) and inspired oxygen pressure, the alveolar (PAO_2_) and arterial oxygen pressure (PaO_2_) are lower compared to sea level. This PaO_2_ decreases even more with age (9, 10) and in subjects with lung disease (6, 11–13). Although the decreased PAO_2_ causes hypoxic pulmonary vasoconstriction, which can increase pulmonary artery pressure at less advanced stages of respiratory disease (14, 15), there are no studies reporting more PH in patients with IPF living at high altitude.

The respiratory and non-respiratory comorbidities of patients with IPF in Latin America are less known and whether they differ between countries in the region. Taking this into account and the fact that there are no comparative studies that have shown more pulmonary hypertension in patients with IPF living at high altitudes, our objective was to describe and compare IPF comorbidities in four Latin American countries and analyze possible differences by altitude.

## Methods

### Participants

Retrospective study in four Latin American countries. Expert groups on interstitial disease from five cities were asked for demographic data, respiratory function tests, echocardiography, and comorbidities of patients with IPF that meet the diagnostic criteria of the 2011 ATS/ERS/JRS/ALAT guidelines (1). The study included patients diagnosed between 2014 and 2018. The cities included and their altitude were: Bogotá, Colombia (2,640 m); Buenos Aires, Argentina (25 m); Mexico City, Mexico (2,240 m); Lima, Peru (150 m), and Trujillo, Peru (34 m). The study was approved by the Research Ethics Committee of the FNC (Approval Number 201902-24111) and the participants were asked for their authorization to be included in the study by signing an informed consent, maintaining the confidentiality of their data.

### Clinical Data and Comorbidities

Clinical records were reviewed to establish the presence of comorbidities at the time of IPF diagnosis. The body mass index (BMI) was used to define obesity (≥30) and underweight (<18.5). Emphysema was defined as the concomitant finding of IPF signs on chest tomography (CT) plus emphysema in the upper lobes. Possible PH was defined by findings in the transthoracic echocardiogram (TE) of systolic pulmonary arterial pressure (sPAP) >36 mm Hg or indirect signs of PH in the absence of other causes of PH: left ventricular systolic dysfunction with ejection fraction <40%, diastolic dysfunction greater than grade I or valvular disease greater to moderate (16). At the time of collecting the information on comorbidities in the clinical records of the patients, it was recorded whether the patients had died. Age, physiology, and comorbidities were used to calculate the TORVAN index, a validated predictive mortality index in IPF (17).

### Pulmonary Function Test

Data of forced vital capacity (FVC), forced expiratory volume in the first second (FEV_1_), FEV_1_/FVC ratio, diffusion of carbon monoxide (DLCO), arterial blood gases, meters walked and oxygen saturation (SpO_2_) during SMWT, were registered. To compare lung function between countries, the reference values for spirometry and DLCO were calculated in all participants using Crapo's equations (18, 19). The alveolar-arterial oxygen tension gradient (A-aPO_2_) was calculated with the simplified alveolar gas equation using the BP of each city.

### Data Analysis

In continuous variables, the assumption of normality was evaluated by the Kolmogorov Smirnov-test and they are presented as means and standard deviation or medians and interquartile ranges. In the qualitative variables, proportions were calculated. The ANOVA-test or the non-parametric Kruskall Wallis-test was used to compare demographic data, pulmonary function tests and TORVAN index among the four countries, and the χ^2^-test was used to compare the proportions.

To evaluate possible differences in pulmonary hypertension due to altitude, participants with TE from the highest cities (Bogotá and Mexico) were compared with those from sea-level cities (Buenos Aires, Lima, and Trujillo). The Student's *t*-test or the Mann-Whitney-test was used for continuous variables, depending on the distribution of the data, and the χ^2^-test for categorical variables. All *p*-values were two-tailed and values <0.05 were considered statistically significant. SPSS version 17 statistical software was used.

## Results

### Participants

Two hundred and seventy-six patients were included, 50 from Argentina, 86 from Colombia, 91 from Mexico and 49 from Peru, with a mean age of 68.7 ± 8.8 years. Hundred percentage had the requested data, except for the TE result, which could not be obtained in 53 patients (19%). In 83.7% of cases, the pattern on chest CT was definitive UIP and in the remaining 16.3%, a surgical biopsy was performed to confirm the diagnosis. Eighty-one percentage of the total sample were men, with a lower percentage in Peru (59.2%) than other countries (*p* < 0.001). Patients from Argentina had a higher BMI than those from other countries (*p* = 0.002). The smoking index was higher in Argentina (*p* < 0.001) and the years of exposure to wood smoke was higher in Peru (*p* < 0.001). The FVC in the total group was 68.1 ± 19.3 with the highest values in the patients from Colombia (*p* < 0.001). The lowest DLCO values were in patients from Colombia and Mexico (*p* < 0.001). The other demographic data and respiratory function tests are in Table 1.

**Table 1:** Participants characteristics and lung function tests.

	**Total**	**Colombia**	**Mexico**	**Argentina**	**Peru**	***p***
	***N* = 276**	***N* = 86**	***N* = 91**	***N* = 50**	***N* = 49**	
Age, years	68.7 ± 8.8	69.7 ± 10.6	67.1 ± 7.7	68.5 ± 8.4	69.8 ± 7.8	0.180
Male sex	225 (81.5)	68 (79.1)	84 (92.3)	44 (88.0)	29 (59.2)	<0.001
BMI, kg/m^2^	26.9 ± 4.5	26.6 ± 3.9	26.6 ± 3.9	29.1 ± 5.0	25.6 ± 5.5	0.002
Lung biopsy	45 (16.3)	13 (15.1)	22 (24.2)	9 (18.0)	1 (2.0)	0.009
Smoking history	163 (59.1)	63 (73.3)	56 (61.5)	39 (78.0)	5 (10.2)	<0.001
Smoking, pack-years	11.0 (2.5–30.0)	7.0 (1.0–30.0)	8.0 (2.3–20.0)	20.0 (12.0–40.0)	6.0 (3.0–30.0)	<0.001
Wood smoke exposure, years	10.0 (9.0–25.0)	7.0 (6.0–30.0)	10.0 (9.0–15.5)	-	20.0 (10.0–30.0)	0.362
FVC, % predicted	68.1 ± 19.3	75.8 ± 16.4	63.0 ± 19.1	66.5 ± 16.4	65.7 ± 22.7	<0.001
FEV_1_, % predicted	72.6 ± 19.9	79.2 ± 18.1	67.1 ± 19.7	71.5 ± 16.3	72.5 ± 22.9	0.001
FEV_1_/FVC, %	84.2 ± 7.8	82.0 ± 8.1	84.3 ± 7.7	84.4 ± 7.3	87.7 ± 6.6	0.001
DLCO, % predicted	47.0 ± 17.8	50.0 ± 13.6	40.4 ± 18.5	52.5 ± 18.7	58.9 ± 23.9	<0.001
PaCO_2_, mmHg	35.4 ± 4.9	34.9 ± 3.3	32.3 ± 4.4	38.9 ± 3.9	42.0 ± 6.2	<0.001
PaO_2_, mmHg	60.2 ± 14.2	52.5 ± 7.7	55.7 ± 9.0	83.2 ± 9.6	72.6 ± 5.5	<0.001
SaO_2_, %	88.3 ± 5.9	86.4 ± 4.9	86.6 ± 6.1	94.9 ± 1.8	92.9 ± 3.2	<0.001
A-aPO_2_, mmHg	15.0 ± 8.9	11.5 ± 7.1	16.6 ± 9.0	17.9 ± 10.5	23.3 ± 6.6	<0.001
**SMWT**						
Distance, m	425.1 ± 119.4	471.8 ± 111.1	419.8 ± 131.3	403.9 ± 97.2	356.1 ± 113.6	0.001
SpO_2_ at the end of the test, %	92.1 ± 3.6	88.9 ± 3.0	92.4 ± 2.6	94.8 ± 2.4	94.7 ± 2.2	<0.001
SpO_2_ end of the test, %	82.8 ± 7.7	77.7 ± 6.1	81.9 ± 6.7	87.4 ± 6.7	89.2 ± 6.0	<0.001

*P = differences between countries. Values as mean ± SD, median (P25–P75) or N (%)*.

*BMI, body mass index; FVC, forced vital capacity; FEV_1_, forced expiratory volume in the first second; DLCO, carbon monoxide diffusing capacity; PaO_2_, arterial oxygen pressure; PaCO_2_, carbon dioxide arterial pressure; HCO_3_, bicarbonate; SaO_2_, oxygen arterial saturation; A-aPO_2_, alveolar-arterial oxygen tension gradient; SMWT, six-minute walking test; SpO_2_, oxygen saturation by pulse oximetry*.

### Comorbidities

**Figure 1:**
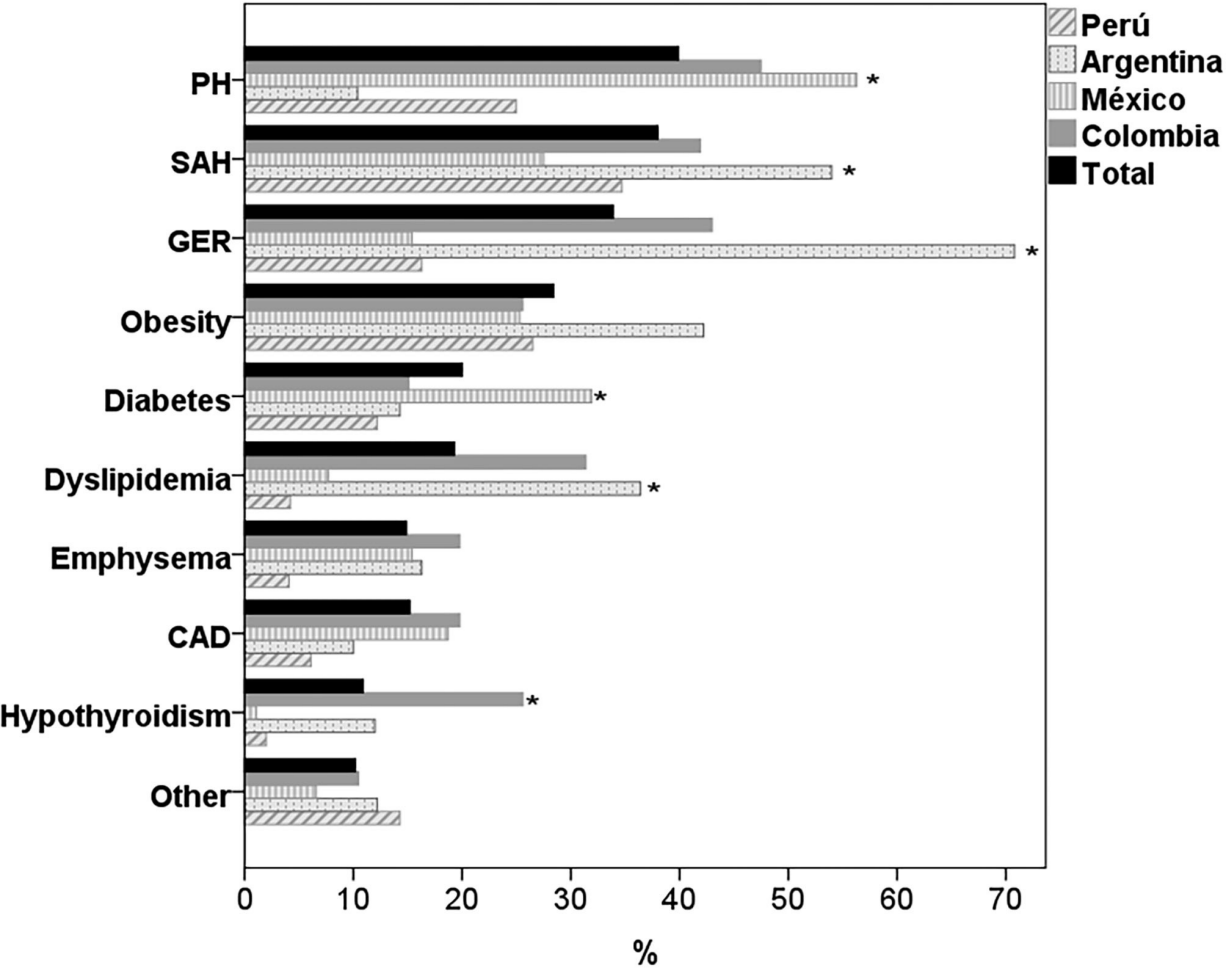
Comorbidities in patients with IPF by country. PH, pulmonary hypertension; SAH, systemic arterial hypertension; GER, gastroesophageal reflux; CAD, coronary artery disease. *p < 0.05 for differences between countries.

The most frequent comorbidities in the four countries were PH, systemic arterial hypertension (SAH), GER and obesity (Table 2). There were significant differences between countries, with a higher prevalence of PH in Colombia and Mexico (*p* < 0.001), of SAH in Argentina (*p* < 0.015), of GER and dyslipidemia in Colombia and Argentina (*p* < 0.001) and of diabetes mellitus (DM) in Mexico (*p* > 0.007). 28.4% of the patients were obese, with no differences between countries (*p* = 0.166) and only 8 subjects (3.0%) of the total sample were underweight (BMI <18.5). Other comorbidities such as coronary artery disease and the presence of emphysema on chest CT were also frequent, with no differences between countries (Figure 1). The presence of cerebrovascular disease, chronic kidney disease, atrial fibrillation, chronic occlusive arterial disease, and lung cancer was documented in <5% of the participants.

**Table 2:** Comorbidities by country.

	**Total**	**Colombia**	**Mexico**	**Argentina**	**Peru**	***p***
	***N* = 276**	***N* = 86**	***N* = 91**	***N* = 50**	***N* = 49**	
Number of comorbidities	2.0 (1.0–3.0)	2.0 (1.0–3.0)	1.0 (0.0–2.0)	2.0 (1.0–3.0)	1.0 (0.0–2.0)	<0.001
Pulmonary hypertension	89 (39.9)	38 (47.5)	40 (56.3)	5 (10.4)	6 (25.0)	<0.001
SAH	105 (38.0)	36 (41.9)	25 (27.5)	27 (54.0)	17 (34.7)	0.015
GER	93 (33.9)	37 (43.0)	14 (15.4)	34 (70.8)	8 (16.3)	<0.001
Obesity	77 (28.4)	22 (25.6)	23 (25.3)	19 (42.2)	13 (26.5)	0.166
Diabetes	55 (20.0)	13 (15.1)	29 (31.9)	7 (14.3)	6 (12.2)	0.007
Dyslipidemia	52 (19.3)	27 (31.4)	7 (7.7)	16 (36.4)	2 (4.2)	<0.001
Coronary artery disease	42 (15.2)	17 (19.8)	17 (18.7)	5 (10.0)	3 (6.1)	0.093
Emphysema	41 (14.9)	17 (19.8)	14 (15.4)	8 (16.3)	2 (4.1)	0.101
Hypothyroidism	30 (10.9)	22 (25.6)	1 (1.1)	6 (12.0)	1 (2.0)	<0.001
Cerebrovascular disease	11 (4.0)	2 (2.3)	4 (4.4)	4 (8.2)	1 (2.0)	0.379
Chronic kidney disease	7 (2.5)	4 (4.7)	1 (1.1)	0 (0.0)	2 (4.1)	0.056
Atrial fibrillation	5 (1.8)	2 (2.3)	0 (0.0)	1 (2.0)	2 (4.1)	0.212
COAD	4 (1.5)	0 (0.0)	1 (1.1)	1 (2.0)	2 (4.1)	0.231
Lung cancer	1 (0.4)	1 (1.2)	0.0 (0.0)	0 (0.0)	0 (0.0)	0.505
TORVAN index, points	16.0 (12.0–19.0)	16.0 (13.0–18.0)	19.0 (16.0–22.0)	13.5 (9.0–18.0)	14.0 (9.0–16.0)	<0.001

*Values as N (%) or median (P25–P75). P: differences between countries*.

*SAH, systemic arterial hypertension; GER, gastroesophageal reflux; COAD, chronic occlusive arterial disease*.

**Figure 2:**
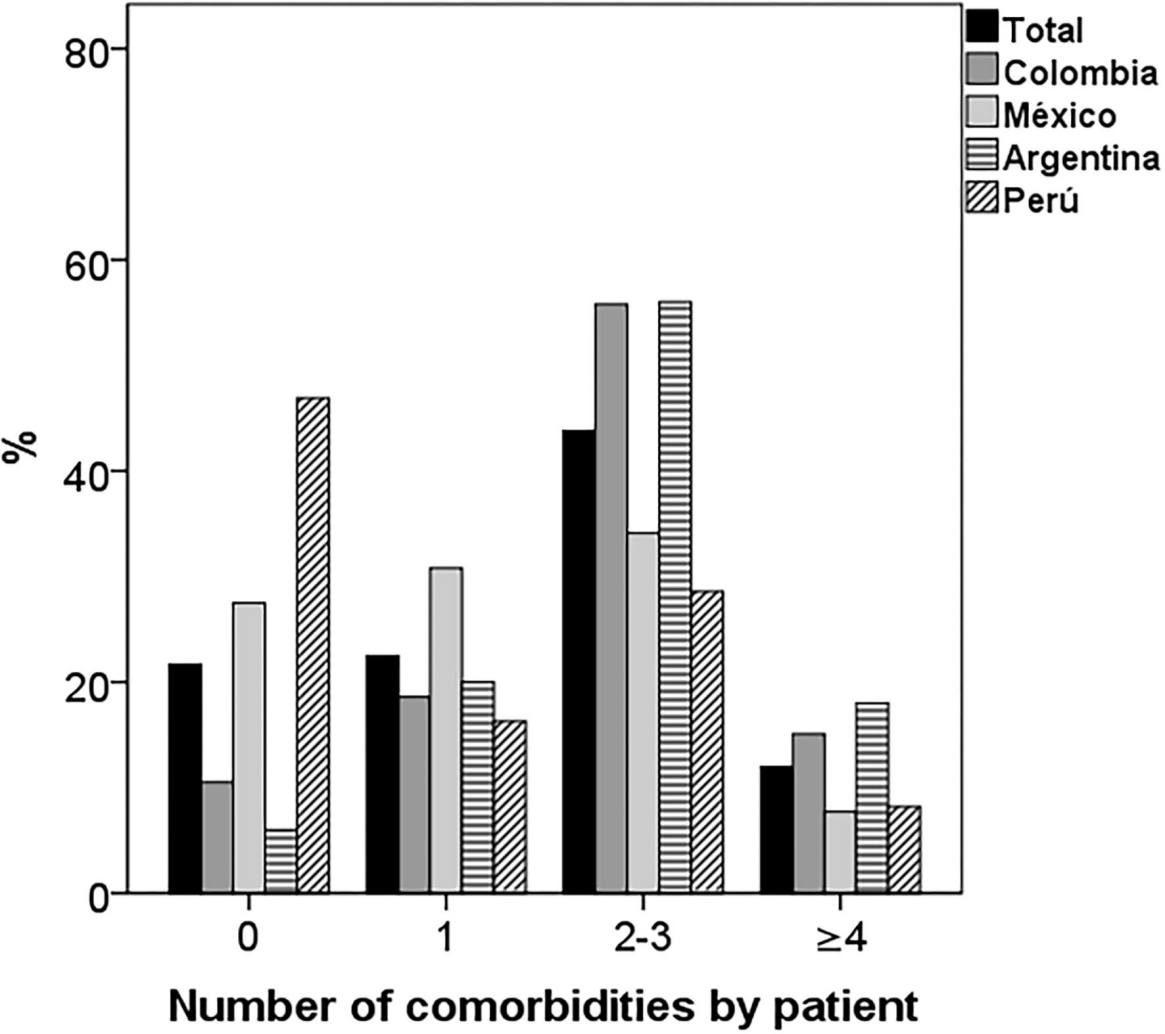
Number of comorbidities per patient by country. There were significant differences in the distribution of comorbidities by country. Most of the patients from Peru and Mexico had one or no comorbidity and those from Colombia and Argentina two or three (*p* < 0.001).

The median number of comorbidities per patient in Colombia and Argentina was two and in Mexico and Peru one (*P* < 0.001; Table 2). In the population studied, there were 60 patients (21.7%) without comorbidities, 62 (22.5%) with one comorbidity, 121 (43.8%) with two to three and 33 (12.0%) with four or more. The country with the highest percentage of patients without comorbidities was Peru (46.9%) and the countries with the highest percentage of patients with two or three comorbidities were Colombia and Argentina (56%) (*p* < 0.001; Figure 2).

**Figure 3:**
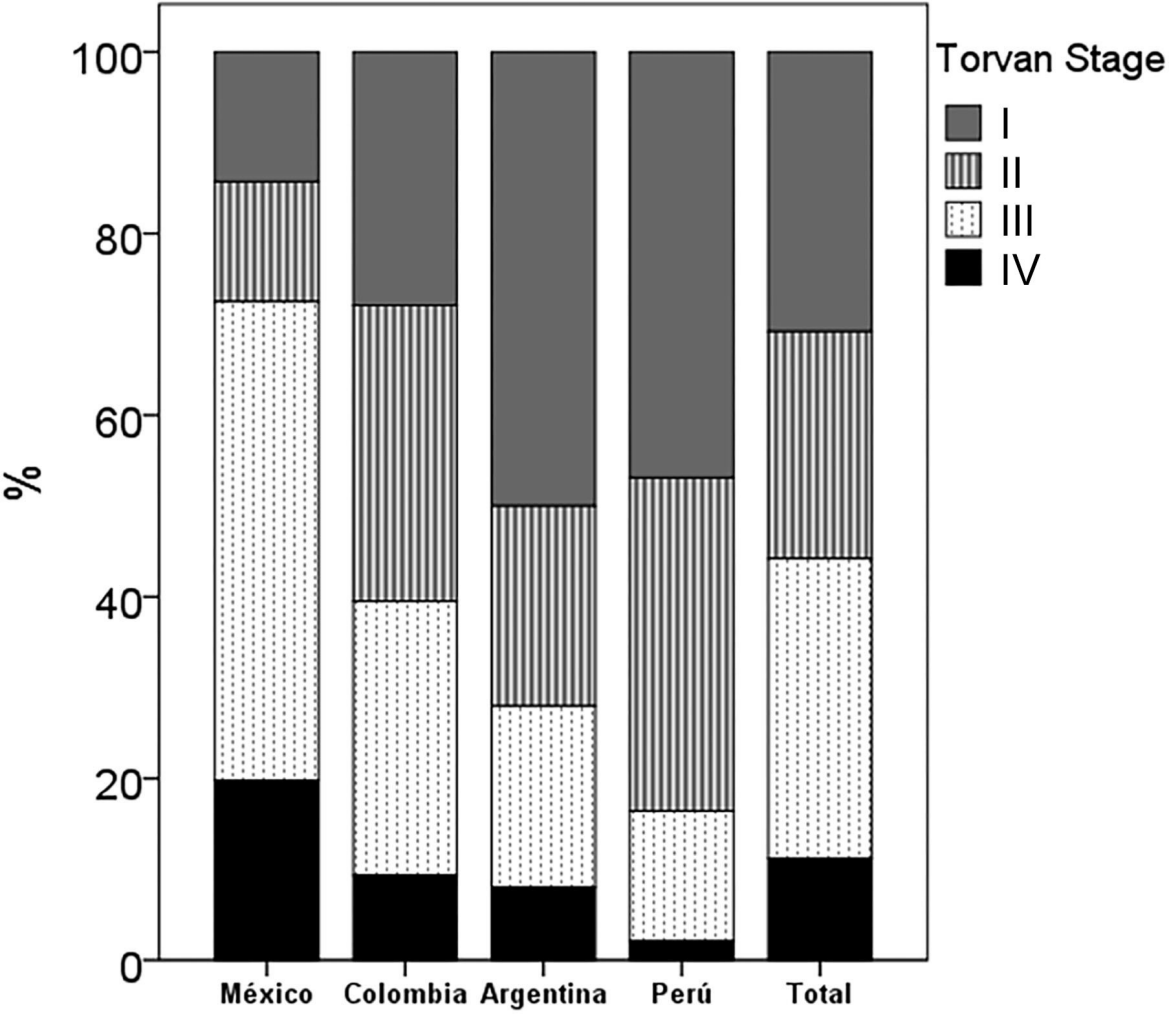
Proportion of patients in the TORVAN states in the total group and in each country. In Mexico, there were more patients in stages III-IV and in Argentina, Colombia and Peru in stages I-II (*p* < 0.001).

At the time of collecting the information, 23.4% of the patients had died. This percentage of deceased patients was significantly higher in Mexico (47.3%) than in Colombia (23.4%), Argentina (10.9%) and Peru (6.1%) (*p* < 0.001). In the total group, the median TORVAN index was 16.0 (12.0–19.0) and it was significantly higher in Mexico than in Colombia, Argentina and Peru (*p* < 0.001; Table 2). Most of the patients in Mexico were classified in TORVAN stages III and V and in Argentina, Colombia and Peru in stages I and II (*p* < 0.001; Figure 3).

### Differences by Altitude

**Figure 4:**
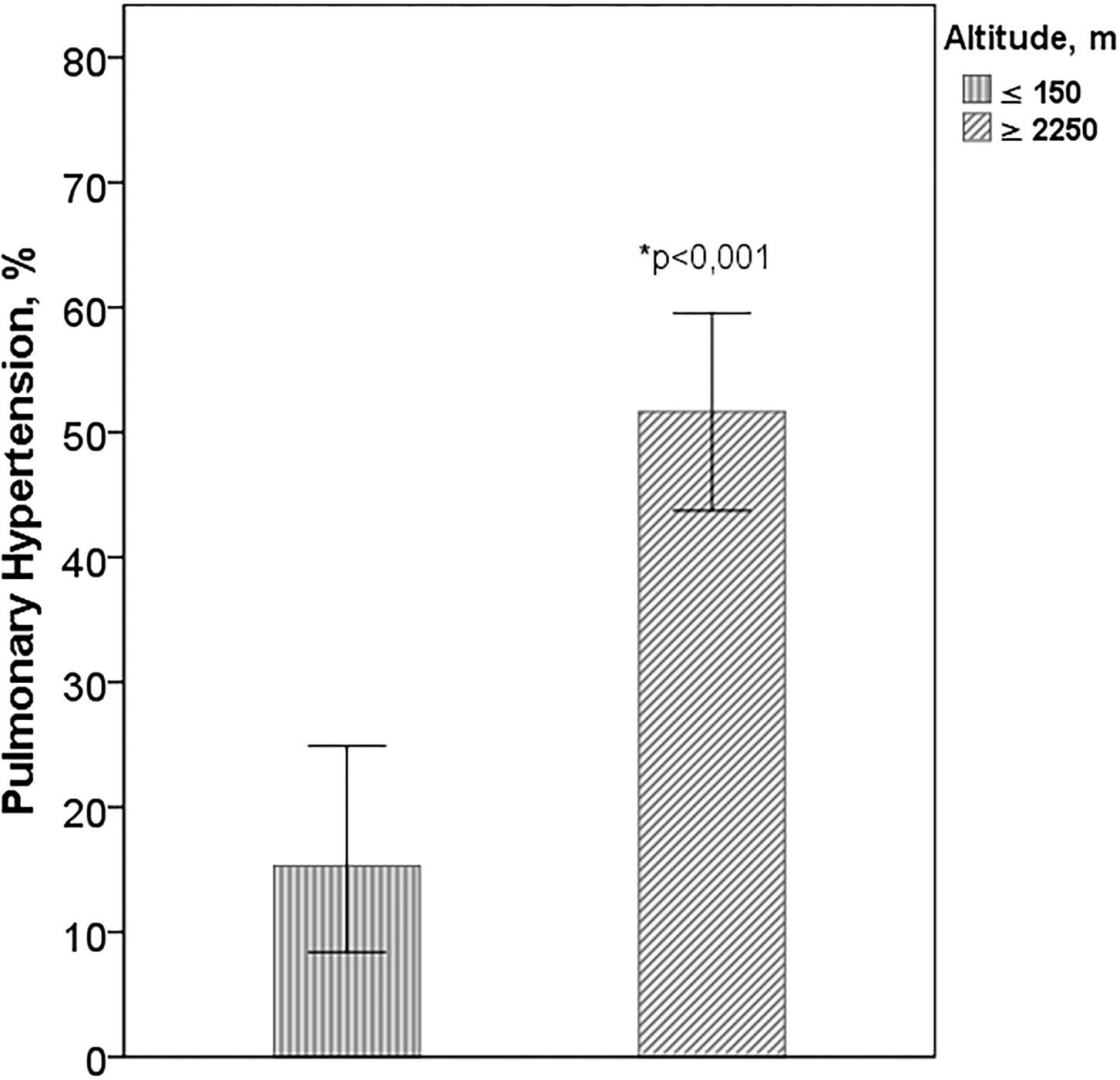
Pulmonary hypertension according to altitude. Altitude: ≥2,250 m Mexico City and Bogotá; ≤ 150 m: Buenos Aires, Lima, and Trujillo. *Difference between the cities of higher altitude with those of sea level.

Of the total of participants, 223 had TE (81%), 73% in cities at sea level and 85% in those of higher altitude. There were no differences in age (*p* = 0.680), sex (*p* = 0.755), or in FVC (*p* = 0.392) between patients with and without TE. The percentage of PH was significantly higher in cities with higher altitude than in those located at sea level, (51.7 vs. 15.3%, *p* < 0.001) (Figure 4). In patients with IPF from Bogotá and Mexico City, PaO_2_, arterial carbon dioxide pressure (PaCO_2_), SpO_2_ at rest and during exercise, and DLCO were significantly lower than in cities at sea level (Table 3). In the cities of higher altitude there was more smoking (67.2 vs. 44.4%, *p* < 0.001), DM (23.7 vs. 13.3%, *p* = 0.038) and coronary heart disease (19.2 vs. 8.1%, *p* = 0.014) and there were no differences in the percentage of patients with emphysema on CT (*p* = 0.103).

**Table 3:** Characteristics of participants with echocardiogram by altitude.

	**Total**	**Altitude ≤150 m**	**Altitude ≥ 2,250 m**	***p***
	***N* = 223**	***N* = 72**	***N* = 151**	
Age, years	68.6 ± 9.1	69.3 ± 8.3	68.3 ± 9.5	0.461
BMI, kg/m^2^	27.2 ± 4.4	27.5 ± 5.5	27.0 ± 3.8	0.504
Pulmonary hypertension	89 (39.9)	11 (15.3)	78 (51.7)	<0.001
sPAP, mmHg	43.5 ± 19.7	31.9 ± 15.7	47.5 ± 19.5	<0.001
FVC, % predicted	68.7 ± 18.3	64.1 ± 17.0	70.7 ± 18.5	0.014
FEV_1_, % predicted	73.1 ± 19.0	70.2 ± 17.6	74.3 ± 19.5	0.140
FEV_1_/FVC, %	84.0 ± 7.9	86.1 ± 7.2	83.0 ± 8.1	0.006
DLCO, % predicted	48.3 ± 17.7	55.0 ± 18.9	46.3 ± 16.9	0.004
PaCO_2_, mmHg	35.9 ± 4.9	40.3 ± 5.1	34.4 ± 3.9	<0.001
HCO_3_, meq/L	23.4 ± 2.9	25.5 ± 3.0	22.6 ± 2.5	<0.001
PaO_2_, mmHg	59.4 ± 15.0	81.3 ± 9.4	52.6 ± 8.1	<0.001
SaO_2_, %	87.9 ± 6.2	94.2 ± 2.7	85.9 ± 5.6	<0.001
A-aPO_2_, mmHg	15.1 ± 9.8	18.0 ± 8.8	13.7 ± 8.7	0.060
**SMWT**				
Distance, m	428.7 ± 121.3	396.7 ± 103.4	448.0 ± 127.8	0.021
SpO_2_ at the end of the test, %	92.1 ± 3.8	95.0 ± 2.4	90.5 ± 3.5	<0.001
SpO_2_ end of the test, %	82.9 ± 7.8	88.3 ± 6.4	79.8 ± 6.7	<0.001

*Values as mean ± SD, or N (%)*.

*P: differences by altitude*.

*BMI, body mass index; sPAP, systolic pulmonary arterial pressure; FVC, forced vital capacity; FEV_1_, forced expiratory volume in the first second; DLCO, carbon monoxide diffusing capacity; PaO_2_, arterial oxygen pressure; PaCO_2_, carbon dioxide arterial pressure; HCO_3_, bicarbonate; SaO_2_, oxygen arterial saturation, A-aPO_2_, alveolar-arterial oxygen tension gradient; SMWT, six-minute walking test; SpO_2_, oxygen saturation by pulse oximetry*.

*Altitude: ≥2,250 m Mexico City and Bogotá; ≤ 150 m: Buenos Aires, Lima, and Trujillo*.

## Discussion

In this study with a significant number of patients, we were able to describe and compare the comorbidities of IPF in four Latin American countries, which contributes to the epidemiological data of this disease in the region. The main results were the differences in comorbidities between the countries and the higher percentage of PH in the subjects residing in the cities of higher altitude.

As expected in IPF, most of the patients were men (81.5%) and with a high percentage of smoking (59.1%), higher than in the general population of these same countries (20–23). The number of comorbidities per patient was lower and the percentage of patients without comorbidities greater than that described in a previous study in Europa (24), although in the total group, 55.8% of the patients had 2 or more comorbidities. As relevant data, there were differences between countries in the number of comorbidities, being significantly higher in Argentina and Colombia.

The most frequent respiratory comorbidities in the four countries were PH and emphysema. The percentage of patients with PH was 39.9%, similar to previous studies. In the systematic review by Raghu (7), the informed prevalence of PH was between 3 and 86%, although most of the data were between 30 and 50%. In 22 (51.2%) of the 43 studies analyzed in this review, the prevalence was estimated by sPAP values in the TE with cut-off points between 35 and 40 mmHg, similar to that used in this study. In the studies in which right catheterization was used, which is considered the gold standard for the diagnosis of pulmonary hypertension, the reported prevalence was between 29 and 46% (25).

We highlight the higher percentage of subjects with PH in the TE in the cities of higher altitude (51.7 vs. 15.3%, *p* < 0.001). The mechanism of hypoxic vasoconstriction with a secondary increase in pulmonary vascular resistance triggered by lower PAO_2_ values at altitude, the pulmonary artery remodeling described in long-term exposure to hypoxia and the erythrocytosis described in altitude in healthy subjects and in patients with respiratory disease (10, 26), could explain the development of PH in these patients (14, 15). Along the same lines of our data, in previous studies in Mexico City and Bogotá, high prevalence of PH have been described in patients with chronic respiratory disease (27, 28).

As expected, there were differences in arterial blood gases between cities of different altitude. In patients from Bogotá and Mexico City, PaCO_2_ was lower, explained by the adaptive mechanism of hyperventilation at altitude (10, 29). Due to the decrease in PAO_2_, PaO_2_ and saturation at rest and during exercise were significantly lower in patients from higher altitude cities. Although the DLCO decrease is a characteristic functional finding of IPF, it was even lower in patients from higher altitude cities, despite having less involvement of the FVC and not having greater emphysema on chest CT, which could be explained due to possible PH in these patients.

Similar to the studies in PH, different definitions have been used to establish the prevalence of emphysema in IPF, such as disease diagnosis codes, questionnaires, pathology findings or CT scan (7). In the studies that have used CT with the definition of the presence or absence of emphysema, without its quantification, the reported prevalence is from 19 to 67% and in those that used the quantification of the extent of emphysema from 8 to 28%. In this study, the prevalence of emphysema was lower (14.9%) and there were no differences between countries, although there were differences in exposure to tobacco and wood smoke. The smoking rate was higher in Argentina and the years of exposure to wood smoke was higher in Peru.

The most frequent non-respiratory comorbidities in the four countries were SAH, GER, obesity, and DM. The prevalence of SAH in these patients was high (38.0%), although it was similar to some series of patients with IPF (7, 24). The highest percentage of SAH was in Argentina, a country with a higher prevalence of this disease in the general population compared to what was described in Colombia, Mexico and Peru (20–23). 28.4% of the patients were obese, similar to that described in other IPF studies (7, 30). Compared with the general population of these countries, this percentage of obesity was higher than that reported in Peru and Colombia, but lower than that described in Argentina and Mexico (20–23).

Using the cut-off point of BMI ≥ 30 to define obesity, there were no significant differences between countries (*P* = 0.166), but there were differences in BMI, which was significantly higher in patients from Argentina (*P* = 0.002). A low percentage of the study patients (3.0%) were underweight, a factor that has been related to a poor prognosis of the disease, as well as weight loss during follow-up (30, 31). The prevalence of DM (20%) was similar to that reported in other studies (10–40%) (7, 32). We highlight that the prevalence of 31.9% of DM in IPF patients from Mexico was significantly higher than the other countries (*p* < 0.001), despite the fact that the prevalence in the general population in the four countries, including Mexico, is <15% (20–23).

The prevalence of GER reported in IPF is highly variable, with values up to 90%, which could be related to the definition used. In the four countries it was 33.9%, but with significant differences between countries, with the highest prevalence in Argentina (70.8%) and the lowest in Mexico and Peru. Hypothyroidism is another comorbidity of IPF associated with higher mortality (33). The prevalence of 10.9% in these patients was similar to that reported in other IPF studies and higher than that of the general population reported in studies from other countries (7, 32, 33).

The other comorbidities included in the study had a low frequency, such as cerebrovascular disease, chronic kidney disease, atrial fibrillation, chronic occlusive arterial disease, valvular heart disease and lung cancer, which were present in <5% of the participants. It is noteworthy that in the subjects of these four countries, the percentage of lung cancer was very low (0.4%) and lower than that reported in the literature (4 to 23%) (7, 34), which is probably explained by the significantly lower incidence of lung cancer in the general population of Latin American countries compared to the United States, Europe and Asia (35, 36). Among the patients from the four countries, there were no differences in age, a factor related to greater morbidity, mortality, and use of health resources in patients with IPF (37). Additionally, aging and smoking are part of the pathophysiology of IPF and other coexisting diseases such as emphysema and lung cancer (8, 38, 39).

Although we were unable to perform a mortality analysis that included comorbidities, we observed differences between countries with a significantly higher percentage of dead patients in Mexico. Similarly, the TORVAN mortality prediction index had the same trend between countries. Patients from Mexico had a significantly higher TORVAN score than in the other countries and most of these patients were classified into TORVAN stages III and IV, unlike patients from Colombia, Argentina, and Peru, who were mostly classified as stage I and II. Taking into account that the patients were of similar age, the differences in TORVAN between countries could be explained by a greater functional compromise (lower DLCO and FVC) and a higher percentage of PH and DM in patients from Mexico than in other countries.

The differences in the frequency of comorbidities, between the study countries and with that described in the literature, could be explained by the differences in the lifestyle and diet of the populations, the history of exposure to tobacco and the prevalence of these comorbidities in general population. Although the study countries belong to the same geographic region, there are important differences between them in the comorbidities reported in national studies of risk factors in the general population (20–23). On the other hand, the differences in comorbidities reported in studies from Europe and the United States can be related to the aforementioned differences in lifestyles, diet and comorbidities in the general population, and probably in the used methodology; as differences in diagnostic methods, the lack of standardized definitions of some comorbidities such as the percentage of extension of emphysema on CT or differences in the diagnostic method used for others such as GER, and the prospective design of several of these studies (7, 25, 40).

As strengths of this work, we highlight that it is the first study in Latin America with a significant number of patients that describes the comorbidities in IPF and the differences between countries, as well as the presence of more pulmonary hypertension in patients with IPF living at altitude. Although this finding could be expected due to the explained pathophysiological mechanisms related to low PAO_2_, hypoxic vasoconstriction, increased pulmonary vascular resistance, pulmonary artery remodeling and erythrocytosis, there are no previous studies that have demonstrated a higher prevalence of PH in patients with IPF who live at high altitudes compared to those who live at sea level. We believe that this study contributes to the knowledge of the clinical behavior of IPF and the epidemiology of this disease in the region. Although the TE is not a confirmatory examination of PH, it is accepted that it is a useful tool to establish a diagnosis of possible PH and, as already commented, most of the studies that have established the prevalence of PH in IPF were based on a methodology similar to that used in this study, which allowed us to compare our results (7). It should be noted that due to the age and functional characteristics of patients with IPF, it is generally difficult to perform right catheterization in these patients.

This study has several weaknesses related to the retrospective design based on the clinical reports of the patients. Unlike prospective studies, the possibility of underreporting comorbidities is greater. In addition, we did not have information on the treatment of comorbidities, which may have an impact on the outcomes of the disease, and on the improvement of the quality of life and symptoms of the patients. It is important to highlight that in some comorbidities, such as GER, the effect of antacid therapy on disease progression and mortality has been studied. Although benefit in these outcomes has been suggested with proton pump inhibitors (41), most studies have not shown an impact on the progression or survival of IPF (42–44).

An important limitation of the study is that we were unable to perform a multivariate analysis of mortality that included comorbidities. Even so, we found significant differences between countries in the percentage of mortality, which correlated well with the result of the TORVAN index. Also, the evaluation of various IPF comorbidities was not possible. First of all, we do not have data on sleep apnea, a comorbidity described in up to 90% of IPF patients (7, 32), which has been associated with greater cardiovascular comorbidity and risk of death due to intermittent nocturnal hypoxemia (45, 46). Another comorbidity not evaluated was pulmonary embolism, an entity related to higher mortality, but with a low prevalence in IPF (7, 8, 32).

The importance of comorbidities in the clinical course of patients with IPF is clearly recognized, so their identification, treatment and management are part of the comprehensive evaluation of these patients (8, 17, 24, 25). In Latin America, prospective studies are required that include all IPF comorbidities, with standardized definitions, which allow evaluating the impact of these comorbidities on clinical outcomes such as disease progression and mortality.

## Conclusion

In this study with a significant number of patients, we were able to describe and compare the comorbidities of the IPF in four LA countries, which contributes to the epidemiological data of this disease in the region. The main results were the differences in comorbidities between the countries and the higher percentage of PH in the patients residing in the cities of higher altitude, a finding that should be validated in future prospective studies.

## Data Availability Statement

The raw data supporting the conclusions of this article will be made available by the authors, without undue reservation.

## Ethics Statement

The studies involving human participants were reviewed and approved by Fundación Neumologica Colombiana Research Ethics Committee. The patients/participants provided their written informed consent to participate in this study.

## Author Contributions

MG-G, ER-Á, and IB-R contributed to the conceptualization and design of the study. MG-G drafted the initial manuscript and guarantor of this work. All authors contributed to data abstraction and analysis, contributed to manuscript writing, and approved the submission of the final manuscript.

## Conflict of Interest

The authors declare that the research was conducted in the absence of any commercial or financial relationships that could be construed as a potential conflict of interest.

## Abbreviations

IPF: idiopathic pulmonary fibrosis
PH: pulmonary hypertension
TE: transthoracic echocardiogram
sPAP: systolic pulmonary arterial pressure
GER: gastroesophageal reflux
SAH: systemic arterial hypertension
CAD: coronary artery disease
DM: diabetes mellitus
CVD: cerebrovascular disease
CKD: chronic kidney disease
COAD: chronic occlusive arterial disease
A-aPO_2_: alveolar-arterial oxygen tension gradient.
